# Role of salivary miRNAs in the diagnosis of gastrointestinal disorders: a mini-review of available evidence

**DOI:** 10.3389/fgene.2023.1228482

**Published:** 2023-06-29

**Authors:** Maria Oana Săsăran, Claudia Bănescu

**Affiliations:** ^1^Department of Pediatrics 3, George Emil Palade University of Medicine, Pharmacy, Sciences and Technology of Târgu Mureș, Târgu Mureș, Romania; ^2^ Genetics Department, Center for Advanced Medical and Pharmaceutical Research, George Emil Palade University of Medicine, Pharmacy, Science and Technology of Târgu Mureș, Targu Mures, Romania

**Keywords:** salivary, miRNA, biomarker, gastrointestinal disorders, pancreatic cancer, gastric cancer

## Abstract

MiRNAs are short, non-coding RNA molecules, which are involved in the regulation of gene expression and which play an important role in various biological processes, including inflammation and cell cycle regulation. The possibility of detecting their extracellular expression, within body fluids, represented the main background for their potential use as non-invasive biomarkers of various diseases. Salivary miRNAs particularly gained interest recently due to the facile collection of stimulated/unstimulated saliva and their stability among healthy subjects. Furthermore, miRNAs seem to represent biomarker candidates of gastrointestinal disorders, with miRNA-based therapeutics showing great potential in those conditions. This review aimed to highlight available evidence on the role of salivary miRNAs in different gastrointestinal conditions. Most salivary-based miRNA studies available in the literature that focused on pathologies of the gastrointestinal tract have so far been conducted on pancreatic cancer patients and delivered reliable results. A few studies also showed the diagnostic utility of salivary miRNAs in conditions such as esophagitis, esophageal cancer, colorectal cancer, or inflammatory bowel disease. Moreover, several authors showed that salivary miRNAs may confidently be used as biomarkers of gastric cancer, but the use of salivary miRNA candidates in gastric inflammation and pre-malignant lesions, essential stages of Correa’s cascade, is still put into question. On the other hand, besides miRNAs, other salivary omics have shown biomarker potential in gastro-intestinal conditions. The limited available data suggest that salivary miRNAs may represent reliable biomarker candidates for gastrointestinal conditions. However, their diagnostic potential requires validation through future research, performed on larger cohorts.

## 1 Introduction

**FIGURE 1:**
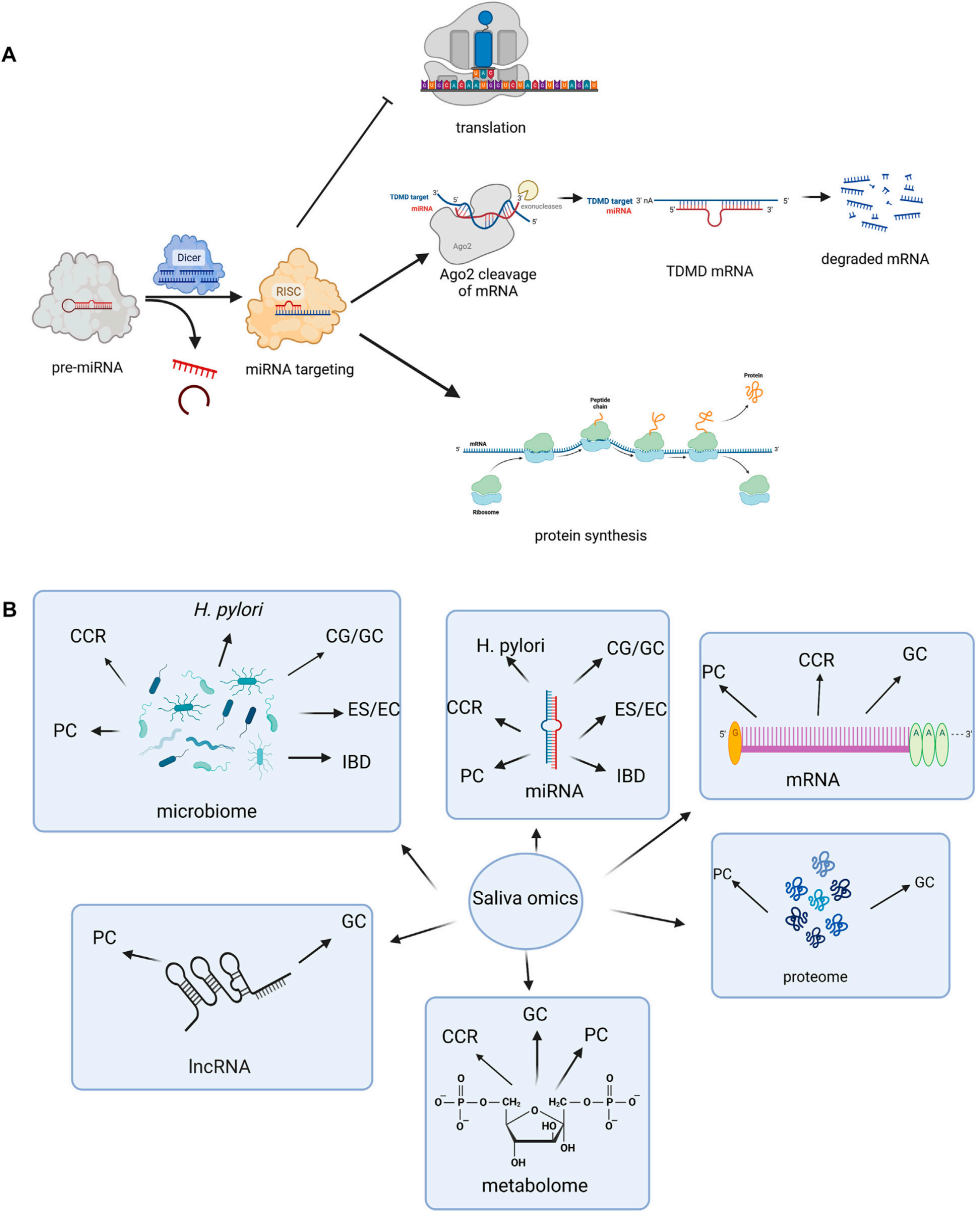
**(A)** Schematic representation of miRNA function (Created with BioRender.com). miRNAs take part in gene expression suppression, which is achieved through translation repression and/or mRNA degradation. Dicer (ribonuclease or helicase with RNase motif) is involved in processing of pre-miRNA (miRNA precursors) into mature miRNA. The main mediator in these processes is represented by the miRNA-induced silencing complex (miRISC) and the ribonucleoprotein (RNP) effector, and later its Ago2 ligand (an important component of RISC that facilitates binding of miRNA). TDMD represents a gene regulation mechanism by which the base-pairing between the miRNA and target RNA is achieved and afterwards their degradation is induced. miRISC-RNP interaction is also able to stimulate gene expression at a posttranscriptional level. Legend of figure: Ago2- Argonaut 2. RISC- RNA-induced silencing complex. TDMD- Target-directed microRNA (miRNA) degradation. **(B)** Salivary biomarkers of gastrointestinal disorders (Created with BioRender.com). Saliva omics are gaining more and more popularity due to their biomarker potential in gastro-intestinal disorders. Besides miRNAs, a few studies also sustain the diagnostic potential of other non-coding RNAs, such as mRNAs and lncRNAs. The prospective of salivary microbiome studies has been highlighted in a variety of gastro-intestinal disorders. Other omics, such as metabolomics and proteome are still scarcely studied, but might represent future non-invasive markers as well. Legend of figure: CCR-colorectal carcinoma. CG-chronic gastritis. EC- esophageal cancer. ES- esophagitis. GC- gastric cancer. H pylori- *Helicobacter pylori*. IBD-inflammatory bowel disease. lncRNA-long non-coding RNA. mRNA-messenger RNA. PC- pancreatic cancer.

MiRNAs represent short, non-coding RNA molecules that are able to regulate gene expression at post-transcriptional level, by targeting the 3′ untranslated region (3′-UTR) of messenger RNAs (mRNAs) (O’Brien et al., 2018; Dexheimer and Cochella, 2020). Specific, stable miRNA interactions with 5′-UTR have also been reported (Broughton et al., 2016). It is important though to acknowledge that miRNAs have multiple mRNA targets, detaining multiple binding sites and thus regulating gene expression in a complex manner (Bartel, 2009; Friedman et al., 2009). Most miRNAs are involved in gene expression suppression, also known as “gene silencing,” which is mediated by the interaction between the miRNA-induced silencing complex (miRISC) and the ribonucleoprotein (RNP) effector (Makarova et al., 2016). Given that miRISC-RNP interaction has been discovered in several cellular compartments, questions were raised about their function extension beyond gene expression modulation (Makarova et al., 2016). At the same time, miRISC-RNP complexes also seem to be able to stimulate gene expression at posttranscriptional levels, with miRNA-mediated effects being highly dependent upon factors such as cellular conditions, RNA sequence, or complementarity degree (Vasudevan, 2012). Thus, miRNAs represent fine tuners of gene expression, as schematically represented in Figure 1A.

MiRNAs are involved in various biological processes, including hematopoietic cell differentiation, cell growth, or epigenetic regulation of malignant transformation-associated pathways (Chen et al., 2004; Wang and Sen, 2011; Wang et al., 2016). Furthermore, miRNAs seem to substantially contribute to normal developmental processes, by modulating trophoblast cell proliferation and apoptosis. Thus, they function as part of the placental development, neuron, astrocyte, muscle cell development, or bone formation (Fu et al., 2013; Ivey and Srivastava, 2015). Their essential role in embryogenesis, cell development, hemostasis and differentiation has also been reported by several studies conducted on different animal species (He et al., 2022; Khanal et al., 2022; Luo et al., 2022). Given their involvement in numerous physiological processes, it is unsurprising that miRNAs and their aberrant expression have been regarded as potential diagnostic biomarkers in a variety of diseases (Hayes et al., 2014; Tüfekci et al., 2014; Huang, 2017). The quest for miRNA signatures is still ongoing, but multiple miRNAs might be validated as future biomarkers in malignant pathologies, viral infections, cardiovascular, renal or neurological disorders (Wang et al., 2016). The possibility of depicting stable, extracellular miRNAs in body fluids such as urine, blood or saliva, and their aberrant expression, which is sometimes similar to tissular analogues, gives the upperhand of potential non-invasiveness to the biomarker status (Huang, 2017; Fellizar et al., 2022).

## 2 Salivary miRNAs

As in all body fluids, circulating miRNAs are found in saliva in lipid/lipoprotein vesicular complexes such as exosomes (Leal-Galvan et al., 2022). Exosomes protect miRNA from degradation, ensuring adequate function of the transported molecular information (Théry et al., 2002; Théry et al., 2009). This translates into stability, circulating, exosomal plasma miRNAs being regarded as highly stable in healthy subjects (Sanz-Rubio et al., 2018). In similar fashion, exosomal salivary miRNAs seem to be easily isolated and quantified within the healthy population, constituting promising, easily available biomarkers (Michael et al., 2010). Some theories suggest that miRNAs could also be produced locally in oral fluids, arousing from apoptosis and cell necrosis. A theory based on the possible existence of transcellular and paracellular transport routes into salivary gland tissues has been issued (Yoshizawa et al., 2013). Moreover, salivary endogenous miRNAs seem to degrade at a slower rate than their exogenous homologues, according to a study which aimed to discover and validate miRNAs in the saliva of healthy subjects (Park et al., 2009).

Saliva collection, although characterized by non-invasiveness, is being subject to various debates. Type of saliva collection has been miscellaneous so far in various reports. Some authors have proposed collection of unstimulated saliva samples, while others have successfully isolated miRNA molecules from saliva collected after citric acid stimulation (Yoshizawa and Wong, 2013; Xie et al., 2015). Saliva composition seems to be influenced by various factors, including meal schedule, diet, time of the day, age or sex (Koopaie et al., 2021). Time of collection and amount of fasting time required prior to collection is still debated and varies from one study protocol to another. Furthermore, administration of agents such as citric acid for stimulation of saliva secretion, prior to its collection, still remains at the liberty of researchers (Setti et al., 2020). Saliva storage tubes vary in volume, with some subjects reporting difficulty in collecting saliva in smaller sized tubes (Urbizu et al., 2023). Isolation can be achieved both from whole saliva and supernatant, obtained after centrifugation, with identification of high resolution miRNA information (Patel et al., 2011). Centrifugation seems to be helpful in case of viscous saliva samples, but the performance rate should be adapted accordingly, in order to avoid mechanical rupture of cellular components (St John et al., 2004).

Due to their non-invasiveness, salivary miRNAs have emerged as promising biomarkers in the diagnosis and prognosis of various conditions, including neoplastic diseases, such as oral cancer, hepatic cancer, breast cancer or pancreatic cancer (Xie et al., 2015; Koopaie et al., 2021; Mariam et al., 2022; Scholtz et al., 2022), as well as inflammatory conditions, including endometriosis, inflammatory bowel disease, periodontitis or eosinophilic esophagitis (Schaefer et al., 2015; Bhardwaj et al., 2020; Lee et al., 2020; Bendifallah et al., 2022). Furthermore, salivary miRNAs seem to present altered expressions in relation to various types of injuries (Hicks et al., 2021; Hicks et al., 2022). Still, the question of similitude between the expression of miRNAs depicted in the saliva and their tissular or circulating homologues is raised. A few studies have tried to simultaneously assess the biomarker role of the same miRNA type in both saliva and blood or saliva and tissue (Nik Mohamed Kamal et al., 2020; Sembler-Møller et al., 2020). A clear view of the reliability of salivary miRNAs in comparison with miRNAs identified among tissue or other body fluids is still subject to future research.

## 3 Salivary miRNAs and gastrointestinal conditions

The quest for biomarkers in gastrointestinal disorders has gained more attention recently and research on the subject bloomed after miRNA-based therapeutics showed a great potential in treatment of various diseases of the gastrointestinal tract (Hossian et al., 2019). Most of the studies conducted so far have focused on the role of miRNA in the diagnosis of gastric cancer, as miRNA expression profile seems to be altered in different stages of Correa’s gastric precancerous cascade (Zheng et al., 2011; Deng et al., 2013; Sugiyama et al., 2016; Lario et al., 2018). However, miRNAs have been usually identified from gastric cellular lines, gastric or esophageal mucosa specimens or depicted in the serum or plasma (Matsushima et al., 2011; Sreedharan et al., 2017; Lario et al., 2018; Ohtsuka et al., 2021; Vasapolli et al., 2021). Studies analyzing the role of salivary miRNAs in gastrointestinal conditions are so far limited in number, but those available so far have shown promising results in terms of delivering non-invasive biomarkers. Therefore, this review aims to highlight available evidence on the role of salivary miRNAs in different gastrointestinal conditions.

### 3.1 Salivary miRNAs, esophagitis and esophageal cancer

Salivary miRNAs distinguished themselves as potential markers of eosinophilic esophagitis. In one pediatric study, in which 56 salivary miRNAs were detected, six different miRNAs (miR-26b-5p, miR-27b-3p, Let-7i-5p, miR-142-5p, miR-30a-5p and miR-205-5p) presented upregulation, quantified through variable importance projection (VIP) scores in subjects with eosinophilic esophagitis. The highest differences between the two study groups were obtained for miR-205-5p, but good sensitivity and specificity parameters were reported for each of the 6 miRNAs (Jhaveri et al., 2023). A similar outcome was described within an adult study, in which miR-4668 emerged as a biomarker candidate that can identify subjects with eosinophilic esophagitis and is correlated with the number of aeroallergens. According to the authors, the same miR-4668 can be used as a response predictor to topical corticosteroids, which suggests that this miRNA could be used as a marker of other allergic disorders as well (Bhardwaj et al., 2020).

More attention has been so far given to the role of miRNAs in the diagnosis and characterization of esophageal cancer. Saliva identifiable miRNAs such as miR-144, miR-451, miR-98, miR-10b and miR-363 might be able to modulate target genes involved in esophageal cancer, according to Du and Zhang (2017). Hoshino et al. describe the high diagnostic performance of miR-1246 in esophageal squamous cell carcinoma, when determined from blood, urine and saliva. Still, variations in salivary miR-1246 levels were found, in relation to saliva collection timing, and its expression differed among the three body fluid samples (Hoshino et al., 2021). On the other hand, plasmatic and salivary miR-21 expressions presented a positive, significant correlation, and comparable diagnostic value in esophageal cancer (Ye et al., 2014). Moreover, high expression of miR-144 was identified in the whole saliva and saliva supernatant of patients with esophageal cancer. Thus, Wu et al. (2013) proposed miR-144 as an early marker of esophageal cancer, with moderate diagnostic performance indices, based on sensitivity and specificity parameters. miRNA identification and expression might though differ between whole saliva and saliva supernatant samples. Hence, Xie et al. found that in subjects with esophageal cancer, three different miRNAs (miR-10b*, miR-144 and miR-451) were upregulated in the whole saliva samples, whereas four of the six identified miRNAs were upregulated (miR-10b*, miR-144, miR-21 and miR-451) within saliva supernatant (Xie et al., 2012; Xie et al., 2013). miR-196a has also been regarded as a suitable salivary biomarker for esophageal squamous cell carcinoma, as its salivary upregulation correlated with the expression augmentation seen in biopsy samples taken from neoplastic tissue, as opposed to the healthy adjacent mucosa (Fendereski et al., 2017).

Given the paucity of studies analyzing the role of salivary miRNAs in the diagnosis of esophageal cancer, and their miscellaneous sensitivity and specificity rates, one meta-analysis was performed and suggested that blood-based miRNAs are more accurate than those identified within saliva samples in detecting esophageal squamous cell carcinoma (Wang et al., 2014). The same conclusion was driven by Li et al., who suggested that both serum and plasma miRNAs are appropriate biomarkers for esophageal cancer (Li et al., 2017). However, Wan et al. claimed that type of body samples from which miRNAs were depicted did not constitute a source of heterogenicity among different miRNA-based studies of esophageal cancer (Wan et al., 2016).

### 3.2 Salivary miRNAs, *Helicobacter pylori* infection and gastric cancer

MiRNAs play a pivotal role in the modulation of host immune response and inflammation triggered by *Helicobacter pylori (H. pylori)* (Săsăran et al., 2021). A few studies have identified an upregulation of tissular and serum miRNAs such as miR-146a, miR-155, miR-127-5p, miR-181 in relation to *H. pylori-*induced chronic gastritis, while others failed to identify a significant variation from healthy subjects in the expression of miR-155 (Chung et al., 2017; Cortés-Márquez et al., 2018; Zabaglia et al., 2018; Mahboobi et al., 2022; Oana et al., 2022). However, only one study so far assessed the role of salivary miRNAs in aiding the identification of *H. pylori* infection, which included children with pulpitis. The authors found a downregulation of miR-204 in pulp tissue, serum and saliva of subjects who were concomitantly infected with *H. pylori,* as opposed to those in whom the bacterial infection was absent. At the same time, expression reduction of miR-204 was correlated with an upregulation of MMP-9, which is known to be influenced by inflammation, possibly caused by both pulpitis and *H. pylori* infection (Zhou and Xu, 2019). Similarly, downregulation of miR-204, in association with upregulation of MMP-9, was reported in patients with *H. pylori*-associated gastric ulcer (Li et al., 2016). Given the proven involvement of miRNAs in the regulation of inflammatory pathways related to *H. pylori* infection, as well as the alteration of miRNA expression in the setting of pre-neoplastic lesions, miRNAs were considered promising biomarkers for early gastric cancer detection, as well as potential therapeutic targets in this malignant setting (Shin and Chu, 2014; Vidal et al., 2016; Khayam et al., 2021). After tissular detection of miRNAs which showed biomarker potential, a search for non-invasive miRNAs, available in accessible body fluids was started (Adami et al., 2019; Zhang et al., 2019; Quirico and Orso, 2020; Lopes et al., 2023). Most of the studies available so far have assessed expression of multiple miRNAs among serum and plasma samples (Lopes et al., 2023). However, only two studies have investigated so far the biomarker potential of salivary miRNAs in gastric cancer individuals. Li et al. discovered and validated a panel of multiple extracellular, salivary miRNAs which were associated with gastric cancer. Among them, a significant downregulation of two miRNA candidates, namely, miR-140-5p and miR-301a, was noted in gastric cancer patients when compared to healthy controls (Li et al., 2018). miRNA panel of two whole saliva- identified miRNAs, miR-140 and miR-301a, might ensure early, non-invasive identification of gastric cancer subjects, according to Kaczor-Urbanowicz et al. However, the authors point out that the performance of these biomarkers, assessed with the help of receiver operating characteristic (ROC) curve, differs among ethnicities (Kaczor-Urbanowicz et al., 2022).

Besides salivary miRNAs, salivary microbiota starts to gain more and more popularity as potential biomarkers of *H. pylori* infection and gastric cancer. The alteration of the gastric microbiome trigerred by *H. pylori* impairs the oral-gut axis and leads to specific changes in salivary microbial community structure (Ji et al., 2020; Chen et al., 2022). In addition, an accumulation of pro-inflammatory bacteria has been noted in relation to gastric cancer, with a particular abundance of *Neisseria* species (Kageyama et al., 2019; Huang et al., 2021). Furthermore, salivary microbiome imbalance has been found in relation to chronic gastric inflammation, even in the absence of *H. pylori* infection (Contaldo et al., 2021).

### 3.3 Salivary miRNAs and differential diagnosis of inflammatory bowel disease (IBD)

The overlapping of clinical symptoms between Crohn’s disease and ulcerative colitis makes an accurate IBD diagnosis challenging, in the absence of endoscopic investigations and microscopic assessment of tissue biopsies. Peripheral blood miRNAs were firstly regarded as non-invasive biomarkers which can distinguish the two main IBD types, with 10 miRNAs presenting a significant upregulation and one miRNA an important expression decrease in the blood of active ulcerative colitis patients, when compared to Crohn’s disease patients (Wu et al., 2011). A panel of salivary miRNAs might be useful in the differential diagnosis of Chron’s disease and ulcerative colitis. In the study of Schaefer et al., expression of miR-101 was altered in the saliva of Chron’s disease patients, whereas miR-21, miR-31, miR-142-3p, and miR-142-5p presented particular expression variation patterns among patients with ulcerative colitis (Schaefer et al., 2015). No other studies have managed so far to evaluate differential expression of salivary miRNAs in IBD patients.

### 3.4 Salivary miRNAs and pancreatic cancer

The search for reliable biomarkers which can identify initial stages of pancreatic cancer has been of crucial importance in the last years, in light of the very low 1-year survival rates that characterize patients with this type of advanced malignancy (Burris et al., 1997). Humeau et al. identified 4 salivary miRNA candidates, namely, miR-21, miR-23a, miR-23b and miR-29c, which were upregulated in pancreatic cancer patients compared to controls, with these miRNAs presenting good sensitivity and excellent specificity. Moreover, miR-23a and miR-23b were already differentially expressed in precursor lesions of pancreatic cancer, while miR-216 was overexpressed in pancreatic cancer subjects when compared to pancreatitis patients (Humeau et al., 2015). Two miRNA candidates, miR-940 and miR-3679-5p, were upregulated and downregulated, respectively, in pancreatic cancer patients when compared to a study group consisting of subjects diagnosed with benign pancreatic tumors and healthy controls. Moreover, these two miRNAs could reliably identify individuals with resectable pancreatic cancer, according to ROC results (Xie et al., 2015). miR-1246 and miR-4644, detected among salivary exosomes, were other two miRNAs which exhibited a significant expression augmentation in pancreatobiliary tract cancer patients when compared to controls. The best area under the curve was calculated for the combined use of the two miRNAs, which suggested their concomitant panel utility for pancreatobiliary tract cancer diagnosis (Machida et al., 2016). On the other hand, another study concluded that whilst miR-1246 is upregulated within the serum and urine of pancreatic cancer patients, its salivary expression in the same individuals did not differ significantly from the one of healthy controls (Ishige et al., 2020).

### 3.5 Salivary miRNAs and colorectal cancer

MiRNAs constitute key players in the pathogenesis of colorectal adenocarcinoma, through their involvement in the regulation of mRNA expression, with eight different miRNAs which act on their mRNA targets being reported as having a differential expression in this malignant setting (miR-141, miR-19a, miR-20a, miR-19b-1, miR-19b-2, miR-16, miR-590 and miR-335) (Wang et al., 2017). Moreover, miR-21 has been particularly intensely studied in colorectal cancer. Circulating miR-21 seems to be a useful tool in the diagnosis of colorectal cancer, whereas tissular miR-21 emerged as a marker of prognosis in subjects with the same malignant condition (Peng et al., 2017). In both plasma and saliva, miR-21 has managed to accurately identify patients with colorectal cancer. Salivary miR-21 presented even higher sensitivity and specificity than plasma miR-21 in supporting colorectal cancer screening (Sazanov et al., 2017). Furthermore, Rapado-Gonzalez et al. proposed a panel of five salivary miRNAs (miR-186-5p, miR-29a-3p, miR-29c-3p, miR-766-3p, and miR-491-5p) for the non-invasive diagnosis of colorectal cancer, which presented significant upregulation in relation to this malignancy (Rapado-González et al., 2019).

## 4 Discussions

Salivaomics, which include proteome, transcriptome, metabolome, microbiome, as well as miRNAs, might represent revolutionary non-invasive tools for the diagnosis of multiple disorders, and especially for the early detection of neoplastic conditions (Wong, 2012). Salivary miRNAs first gained attention as biomarkers of oral pathologies, including periodontal disease or pre-neoplastic lesions or malignancies of the oral cavity (Martina et al., 2020; Kapoor et al., 2021; Manzano-Moreno et al., 2021). Non-coding RNAs, including miRNAs, circular RNAs (circRNAs) and long non-coding RNAs (lncRNAs) have been known to be involved in several important hallmarks of gastrointestinal cancers such as esophageal, gastric, pancreatic, colorectal cancer or hepatocellular carcinoma (Dragomir et al., 2020). Still, data on the biomarker utility of salivary miRNAs in conditions of the gastrointestinal tract is scarce, but research is continuously expanding. Igaz et al., who provided an update regarding the recent reported utility of fecal, biliary, salivary and urinary miRNAs, acknowledge the potential of these biomarkers in revolutionizing early diagnosis of malignancies of the gastrointestinal tract, but at the same out point out towards the need of spreading research on larger cohorts and using uniform methodological approaches (Igaz and Igaz, 2015). Through this review, we aimed to highlight current evidence available regarding the use of salivary miRNAs in various gastrointestinal pathologies. An overview of current studies adhering to our review objective, which suggested that miRNAs might serve as biomarker candidates in various types of conditions, has been provided through Table 1. In addition to salivary miRNAs, a synopsis of other, currently available salivary biomarkers in gastro-intestinal conditions has been provided through Figure 1B, based on recent literature data (Zhang et al., 2010; Xiao et al., 2016; Wang et al., 2017; Asai et al., 2018; Bel’skaya et al., 2020; Deutsch et al., 2020; Xu and Jiang, 2020; Huang et al., 2021; Eftekhari et al., 2022; Koopaie et al., 2022; Patel et al., 2022; Lopes et al., 2023).

**TABLE 1:** Synopsis of studies that assessed the role of salivary miRNAs in gastrointestinal disorders.

Reference (author, year)	Gastrointestinal condition	Type of study	Population and study group assignment	Salivary sample type	miRNA outcome
Jhaveri et al. (2023)	Eosinophilic esophagitis	Case-control study	150 pediatric patients:	whole saliva	↑ miR-26b-5p, miR-27b-3p, Let-7i-5p, miR-142-5p, miR-30a-5p, miR-205-5p in eosinophilic esophagitis patients
			• 50 patients with eosinophilic esophagitis		
			• 100 healthy controls		
Du and Zhang (2017)	Esophageal cancer	Case-control study	10 patients:	whole saliva	↑ miR-144, miR-10b, miR-451, and miR-196a in patients with esophageal cancer
			• 7 patients with esophageal cancer		
			• 3 healthy controls		
Hoshino et al. (2021)	Esophageal cancer	Case-control study	122 patients:	Saliva supernatant	miR-1246 did not present significant expression variation between the two study groups
			• 72 patients with esophageal cancer		
			• 50 healthy controls		
Ye et al. (2014)	Esophageal cancer	Case-control study	150 patients:	whole saliva	↑ miR-21 in esophageal cancer patients
			• 100 patients with esophageal cancer		
			• 50 healthy controls		
Wu et al. (2013)	Esophageal cancer	Case-control study	100 patients:	whole saliva and saliva supernatant	↑ miR-144 in both whole saliva and saliva supernatant of esophageal cancer patients
			• 50 patients with esophageal cancer		
			• 50 healthy controls		
Xie et al. (2013)	Esophageal cancer	Case-control study	58 patients:	whole saliva and saliva supernatant	↑ miR-10b*, miR-144 and miR-451 in whole saliva samples of esophageal cancer patients
			• 39 patients with esophageal cancer		↑ miR-10b*, miR-144, miR-451 and miR-21 within saliva supernatant of esophageal cancer patients
			• 19 healthy controls		
Xie et al. (2012)	Esophageal cancer	Case-control study	48 patients:	saliva supernatant	↑ miR-21 in esophageal cancer patients
			• 32 patients with esophageal cancer		
			• 16 healthy controls		
Fendereski et al. (2017)	Esophageal cancer	Case-control study	10 patients in whom saliva was collected:	whole saliva	↑ miR-196a in esophageal cancer patients
			• 5 patients with esophageal cancer		
			• 5 healthy controls		
Zhou and Xu (2019)	*Helicobacter pylori* infection and pulpitis	Case-control study	45 pediatric patients:	saliva supernatant	↓ miR-204 in children with pulpitis and *H. pylori* infection
			• 26 patients with pulpitis and *H. pylori* infection		
			• 19 patients with pulpitis without *H. pylori* infection		
Li et al. (2016)	*H. pylori* associated gastric ulcer	Case-control study	75 patients:	whole saliva	↓ miR-204 in patients with *H. pylori* associated gastric ulcer
			• 46 patients with *H. pylori* associated gastric ulcer		
			• 29 healthy controls		
Li et al. (2018)	Gastric cancer	Case-control study	20 individuals in whom saliva samples were collected for miRNA profiling:	whole saliva	↓ miR-140-5p and miR-301a in gastric cancer patients
			• 10 patients with *gastric cancer*		
			• 10 healthy controls		
Kaczor-Urbanowicz et al. (2022)	Gastric cancer	Case-control study	100 patients:	saliva supernatant	↓ miR-140-5p and miR-301a-3 in gastric cancer patients
			• 50 patients with gastric cancer		
			• 50 healthy controls		
Schaefer et al. (2015)	Crohn’s disease and Ulcerative colitis	Comparative study	15 subjects in whom saliva was collected:	whole saliva	↑ miR-101 in Crohn’s disease patients
			• 5 patients with Crohn’s disease		↑ miR-21, miR-31 and miR-142-3p in ulcerative colitis patients
			• 5 patients with Ulcerative colitis		↓ miR-142-5p in ulcerative colitis patients
			• 5 healthy controls		
Humeau et al. (2015)	Pancreatic cancer and pancreatitis	Pilot study	17 patients:	saliva supernatant	↑ miR-21, miR-23a, miR-23b and miR-29c in pancreatic cancer patients compared to controls
			• 7 patients with pancreatic cancer		↑ miR-216a in pancreatic cancer compared to pancreatitis
			• 4 patients with pancreatitis		↑ miR-23a, miR-23b in pancreatic cancer precursor lesions compared to controls
			• 2 patients with intraductal papillary mucinous neoplasia		
			• 4 patients with unrelated digestive diseases (control group)		
Xie et al. (2015)	Pancreatic cancer	Case-control study	100 subjects:	saliva supernatant	↑ miR-940 in pancreatic cancer compared to healthy controls and benign pancreatic tumors
			• 40 patients with pancreatic cancer		↓ miR-36795p in pancreatic cancer compared to healthy controls and benign pancreatic tumors
			• 20 patients with benign pancreatic tumors		
			• 40 healthy controls		
Machida et al. (2016)	Pancreato-biliary tract cancer	Case-control study	25 subjects:	whole saliva	↑ miR-1246 and miR-4644 in pancreato-biliary tract cancer patients miR-4306 did not vary significantly between the two study groups
			• 12 patients with pancreato-biliary tract cancers		
			• 13 healthy controls		
Ishige et al. (2020)	Pancreatic cancer	Case-control study	71 subjects:	saliva supernatant	Salivary miR-1246 expression did not differ significantly between the two study groups
			• 41 patients with pancreatic cancer		
			• 30 healthy controls		
Sazanov et al. (2017)	Colorectal cancer	Case-control study	68 subjects:	saliva supernatant	↑ miR-21 in colorectal cancer patients
			• 34 patients with colorectal cancer		
			• 34 healthy controls		
Rapado-González et al. (2019)	Colorectal cancer	Case-control study	107 subjects:	whole saliva	↑ miR-1865p, miR-29a3p, miR-29c3p, miR-7663p and miR-4915p in colorectal cancer patients
			• 51 patients with colorectal cancer		
			• 19 patients with colon adenomas		
			• 37 healthy controls		

Legend: H. pylori—Helicobacter pylori, miR—micro RNA.

As presented within the former chapters, most salivary-based miRNA studies available in the literature that focused on pathologies of the gastro-intestinal tract have been conducted on patients with pancreatic cancer. One meta-analysis which included the formerly described studies revealed that salivary miRNAs facilitate diagnosis of pancreatic cancer, with good performance indices such as pooled sensitivity, specificity, positive and negative likelihood ratios and diagnostic odds ratio. Hence, these have been proposed as biomarkers of early pancreatic cancer (Koopaie et al., 2021). The discriminatory power of salivary miRNAs in the detection of esophageal cancer cannot be foreseen, in spite of only a few available studies (Xie et al., 2012; Xie et al., 2013; Wu et al., 2013; Ye et al., 2014; Fendereski et al., 2017). The use of non-blood based liquid biopsies for timely detection of gastric cancer is another hot topic of current research, but the small number of available studies still raise the need for further research and validation (Lopes et al., 2023). Particular beneficial results could be obtained from the identification of initial stages of Correa’s cascade, which so far has been accomplished through plasmatic and tissular miRNAs (Lario et al., 2018; Kim et al., 2020). In colorectal cancer, the use of fecal miRNAs has so far been considered the ultimate non-invasive screening method, with only a few studies showing the biomarker characteristics of salivary miRNAs (Link et al., 2010; Sazanov et al., 2017; Rapado-González et al., 2019). Some studies offer though encouraging results, in spite of their unicity on the subject. For example, one recent study proved for the first time that salivary miRNA signatures could be used in the early identification of hepatocellular carcinoma (HCC). After identifying an impressive number of 4,565 precursor and mature miRNAs, the authors found that 283 of these were downregulated in patients with HCC (Mariam et al., 2022). Hence, abundance of miRNA candidates in the saliva calls for expansion of future research, in light of the limited data available.

In conclusion, salivary miRNAs may represent reliable biomarker candidates for gastrointestinal conditions. Their diagnostic potential has so far been underlined by a limited number of studies, but future research assessing salivary identifiable miRNAs, performed on larger cohorts, might validate or infirm their biomarker status in a multitude of gastrointestinal disorders, particularly in the early identification of malignancies.
